# A survey of retaining faculty at a new medical school: opportunities, challenges and solutions

**DOI:** 10.1186/s12909-018-1330-z

**Published:** 2018-09-25

**Authors:** Fauzia Nausheen, Mukesh M Agarwal, John J Estrada, Dhammika N Atapattu

**Affiliations:** Department of Medical Education, California University of Science and Medicine - School of Medicine, 217 E. Club Center Drive, San Bernardino, CA 92408 USA

**Keywords:** Faculty, Retention, Development, Promotion, Attrition

## Abstract

**Background:**

At well-established academic university settings, retaining faculty remains a pressing challenge due to competing market forces, decreasing institutional support, and changing personal expectations. There is a paucity of information about the difficulties faced by new medical schools to maintain their academic workforce.

The objective of this study was to determine the challenges facing the faculty at a newly developed medical school.

**Methods:**

Twelve founding faculty were surveyed anonymously by a 32-item questionnaire. Their responses were independently analyzed by three researchers.

**Results:**

The views of the faculty were categorized into in four inter-related themes: personal, support, institutional, and environmental. The constant sources of satisfaction among faculty were higher academic rank (75%), harmonious inter-collegial relationships (74%), healthy pecuniary rewards (58%), better professional growth (58%) along with greater autonomy, administrative independence, minimum groupism and excellent team work. Poor opportunities for promotion (68%), reduced support for scholarly activities (67%) and unsatisfactory support from the administration (55%) were detrimental to retaining faculty.

**Conclusion:**

By addressing specific issues facing its staff, every new medical school will not only manage to retain its academic faculty but also be able to attract well qualified academic staff from established medical institutions worldwide.

## Background

Medical faculty, physicians and scientists remain the cornerstone of any academic medical institution. Thus, a low faculty turnover is crucial for any medical school to function smoothly. To retain its workforce, experts recommend exploring the challenges involving faculty retention and implementing professional programs to resolve them [1]. There are differences in gender, race, ethnicity, rank, and specialty affecting long term faculty retention. Furthermore, medical schools also face significant financial losses due to faculty attrition; for example, the average cost of replacing a generalist can amount up to $115,554 while the cost of replacing a specialist may add up to a whopping $286,503 [2]. In short, the cost of losing faculty for any organization is considerable.

In USA, there are many new medical schools being established. According to the Liaison Committee of Medical Education (LCME), in 2017, there were 8 new applicant medical schools. California University of Science and Medicine - School of Medicine (CUSM - SOM) is a not-for-profit private allopathic medical school located at Colton, California, serving the Inland Empire region of Riverside and San Bernardino counties of Southern California. Its mission is to advance the art and science of medicine, through innovative medical education, research, and compassionate health care in an inclusive environment that promotes critical thinking, creativity, integrity and professionalism. It will specifically admit qualified students from the Inland Empire region who in-turn will serve the region which already has a low physician population. Initiated in 2014, currently it is undergoing the accreditation process by the LCME. The school plans to admit 60 students for its inaugural class with a yearly increment of 30 students to reach a maximum of 120 students per year.

Most studies addressing faculty retention problems are limited to established academic health institutions. However, these issues may or may not apply to newly established medical schools. There are many differences between developed and developing medical schools; new schools generally group faculty members into a single department of medical education that emphasizes curriculum design, development and implementation to comply with accrediting agencies. The founding faculty may find it harder to keep their competitiveness in terms of research, teaching and service due to a) a paucity of opportunities for basic science research (e.g., no well-established laboratory), b) lack of students and c) poor liaison with hospitals, respectively. Due to the many new medical school being established and many more on the horizon, the issues regarding retention faced by the faculty at such institutions are worth addressing.

Data from the American Association of Medical Colleges (AAMC) confirms that 38% of faculty overall and 43% of first-time assistant professors leave academic medicine within a 10-year period; first-time assistant professors with both MD and PhD degrees were more likely to switch medical schools. The attrition rates are higher for faculty who were a) female, b) non-white and c) with an MD degree [3, 4]. Most studies on faculty retention focus on faculty dissatisfaction; however, there are few comprehensive studies addressing the importance of quality of life, support for scholarly work, faculty responsibilities, faculty mentoring, and faculty participation in governance. Most published data can be divided into two categories: a) reasons and predictors for faculty attrition and b) institutional mechanisms to enhance retention. Several studies explore the first two factors, and virtually none describe prospective development and retention programs to solve the problems of faculty attrition. In one retrospective study, the reasons for departure included lack of advancement opportunities, salary concerns, and personal/family issues. Salary was the primary concern for clinical (vs. research) faculty [5]. Other studies have reported increased levels of dissatisfaction due to the institutional culture [6, 7], distress and burnout, unethical behavior in research and aging [8, 9]. For early predictors of attrition, one study found factors such as poor leadership by the department chair, low engagement of faculty in the affairs of the school, and lack of institutional support for scholarly activity [10]. Cultural un-relatedness, moral distress, lack of engagement, and low institutional support were cited as predictors of attrition by over 2000 faculty members at an academic health center [11]. Prevailing factors such as difficulty balancing work and family, inability to comment on performance of institutional leaders, absence of development programs, lack of recognition of clinical care, and absence of an ‘academic community’ were major concerns expressed by a large cohort of faculty. Interestingly, faculty members of interdisciplinary centers were less likely to leave an academic health center [12].

The objectives of this study were to a) determine the needs of founding faculty and, b) address challenges facing faculty retention at a newly developed non-tenure granting medical school.

## Methods

An anonymous survey (of 32 questions) was constructed to explore faculty concerns regarding early attrition and long-term retention. Questions explored faculty perception of the work environment, factors that would hinder and/or facilitate their long-term commitment to the institution, and the support provided to best contribute to the mission and vision of the new medical school. To develop the questionnaire, the data from StandPoint Surveys (AAMC) was explored and important concepts were reviewed by few senior faculty members [13]. All questions were vetted for inconsistency and ambiguity, after which they were modified or removed, and the final questionnaire was developed.

This questionnaire was distributed to all the founding faculty (*n* = 12) who had been employed for over one year. No demographic information (e.g. age and sex) other than academic rank was obtained. The answers of the respondents were classified according to a five-point Likert-scale: very dissatisfied, dissatisfied, neither satisfied nor dissatisfied (neutral), satisfied and very satisfied. Basic statistics were used to determine the following: 1) the percentage of respondents giving the same specific answer 2) the ranking average for each answer choice to determine the most preferred overall [14, 15]. The answers were independently analyzed by three faculty researchers. The study was anonymous and confidential. All participants were aware that the data was a research survey with a potential to be published. The study protocol was approved by the local Institutional Review Board.

## Results

**Fig. 1:**
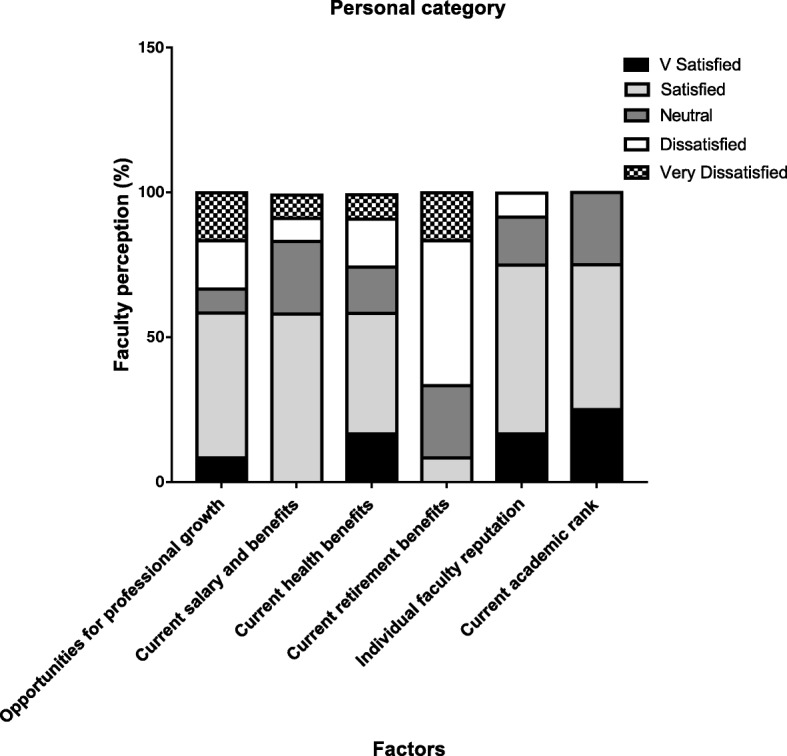
Illustrates the degree of satisfaction of 12 faculty members (as a percentage) on personal category. Level of satisfaction was divided into 5 discrete categories as indicated in the key

The response rate was 100%. Seven members were full professors while five held the rank of an associate professor. At the time of the survey, there were no junior faculty. The responses were grouped into the following inter-related categories: 1) Personal: monetary and health, 2) Support: administration and leadership 3) Institutional: promotion and retention and 4) Environment: regional and societal. These categories were developed to make it easier to analyze the data, and more importantly to develop an action plan for faculty development and retention as deemed by the office of the Dean for Faculty Affairs. Personal category (Fig. 1) showed that majority of the faculty were either satisfied (50%) or very satisfied (8%) with their professional growth opportunities and current salary and health benefits (58%); however, most of the faculty were dissatisfied (50%) or very dissatisfied (17%) with their retirement benefits. Majority of the faculty (75%) expressed high to normal level of satisfaction with the professional reputation of their colleagues and their academic ranking.

**Fig. 2:**
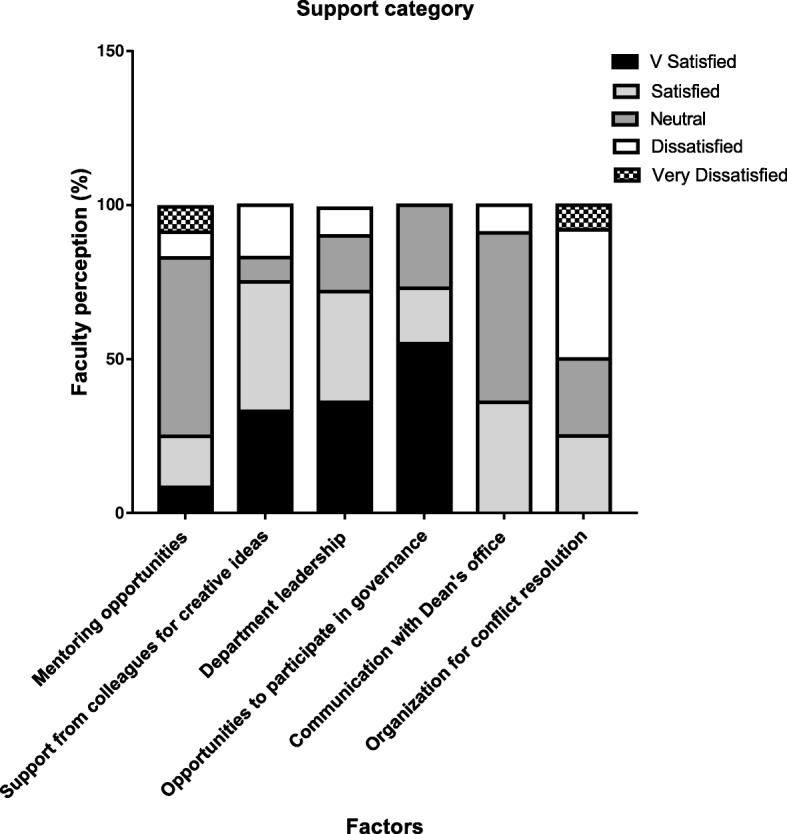
Illustrates the degree of satisfaction of 12 faculty members (as a percentage) on support category. Level of satisfaction was divided into 5 discrete categories as indicated in the key

The Support category (Fig. 2) illustrates that 58% faculty members were neutral and 17% were not satisfied with mentoring opportunities but were content (satisfied and very satisfied) with the collegial support for creative ideas (75%). More than two-third of the faculty were satisfied and very satisfied with the department leadership (72%) and opportunities to participate in governance (73%). Communicating with the Dean’s office was considered acceptable (36% satisfied and 55% neutral) while organization for conflict resolution (50% were dissatisfied and very dissatisfied) was yet another problem.

**Fig. 3:**
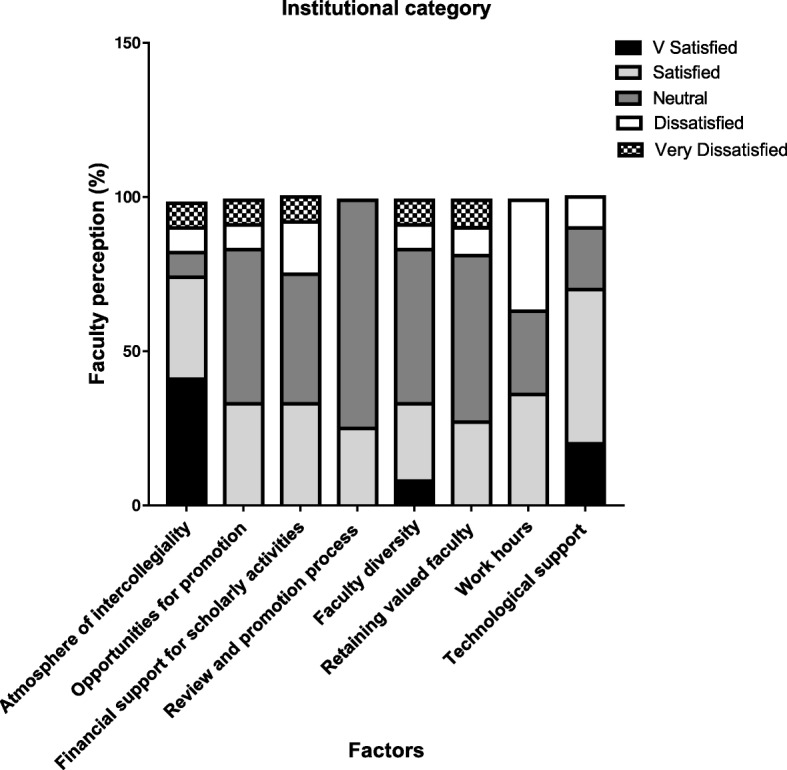
Illustrates the degree of satisfaction of 12 faculty members (as a percentage) on institution category. Level of satisfaction was divided into 5 discrete categories as indicated in the key

The Institution category (Fig. 3) indicated that most of the faculty were satisfied with the inter personal and collegial atmosphere (41% were very satisfied and 33% were satisfied). Half of the faculty were indifferent with opportunities for promotion and so was with the promotion process itself.

Other areas where the faculty were ambivalent were financial support for scholarly activities, diversity, retaining faculty and faculty working hours. Satisfaction was high (70%) with technological support provided by the Information Technology staff.

**Fig. 4:**
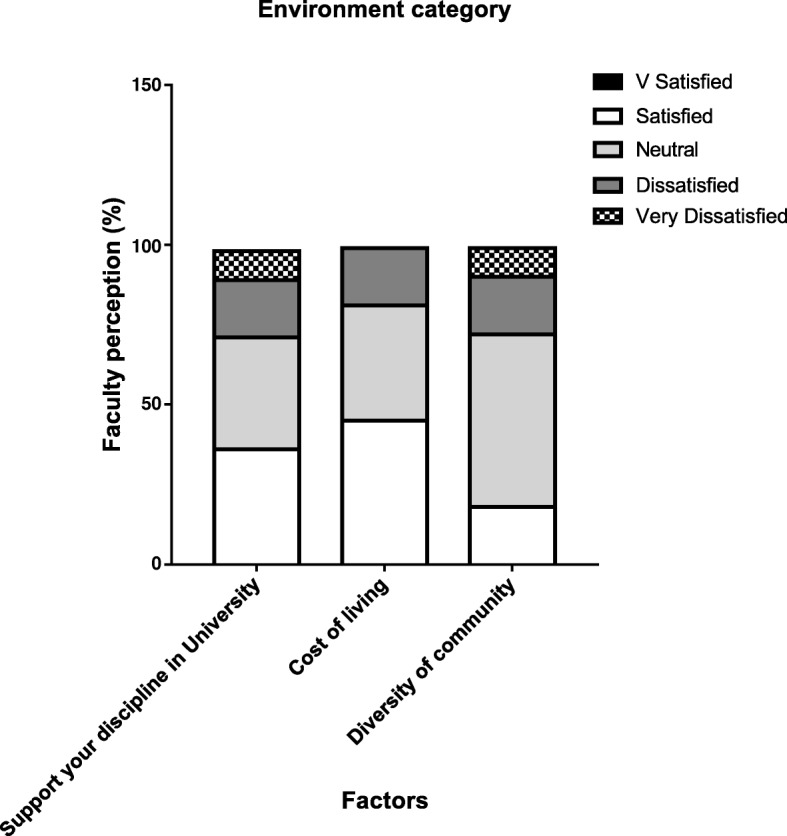
Illustrates the degree of satisfaction of 12 faculty members (as a percentage) on environment category. Level of satisfaction was divided into 5 discrete categories as indicated in the key

Environment category (Fig. 4) confirmed that most of the faculty were neutral or satisfied with opportunities to support their disciplines within the university (71%), cost of living (81%) and with diversity of professional community (72%).

In general, personal category questions were rated more important followed by Support category and Institutional category. Inter-collegiality (74%), current academic rank (75%), individual faculty reputation (75%), department leadership (72%), opportunities to participate in governance (73%) and support from colleagues for creative ideas (75%) ranked highly among respondents of this survey. We also compared our data with a larger study performed by AAMC on factors effecting faculty retention which involved 6 medical schools in the US (Table 1).

**Table 1:** Comparison of survey results between AAMC and CUSM-SOM

Themes (Factors)	AAMC (%)	CUSM – (SOM %)
Medical school governance (Adequacy of communication from Dean’s office)^a^	83	75
Collegiality (Inter-collegiality)^a^	72	74
Compensation/Benefits (Salary/Benefits)^a^	62	58
Growth Opportunities (Opportunities for professional growth)^a^	61	58
Promotion equality (Opportunities for promotion)^a^	72	33
Relationship with Supervisor (Satisfaction with mentoring as mentor/mentee)^a^	70	25
Recruitment & Retention (Efforts to hire high quality faculty)^a^	63	41
Department Governance (Department leadership)^a^	63	72
Job satisfaction (Work hours, adequacy for time for research and degree of autonomy)^a^	83	36

^a^CUSM – SOM themes

## Discussion

New medical schools, like their established counterparts, face numerous challenges like faculty retention. Many findings of this study were to be expected intuitively, however, there were a few surprises. This information should help to preempt some of the problems facing charter schools (i.e., new schools with a curriculum and educational philosophy different from the other medical schools in the system).

Most similar surveys involve established medical schools. It is useful to compare our data with similar studies addressing faculty retention. Results from 6 medical schools are summarized in an AAMC report [16]. The collegiality, benefits, medical school governance, growth opportunities were similar in both established and new medical schools. However, opportunities for promotion, relationship with supervisor (mentoring of faculty as mentor/mentee), job satisfaction (work hours, adequacy for time for research and degree of autonomy) and retention/ recruitment (efforts to retain high hire quality faculty) were much less at the new medical school (Table 1). The reasons for the differences are easy to decipher. Opportunities for promotion are limited in new schools as systems for promotion are evolving. An absence of junior faculty leaves few opportunities for the senior faculty to mentor, nevertheless priorities towards accreditation overshadow the will to mentor the junior faculty. Efforts to hire high quality faculty are harder and job satisfaction is less in new schools due to non-LCME accreditation, absence of tenure and a lack of start-up funds for research.

It is worthwhile to compare our school to other new schools as well as older established schools. The similarities and differences between these categories of schools will help to view the results of this survey in a more global context. Compared to well-established medical schools, the newer schools have a) limited funding, b) no branding (unless they are a part of a well-established university), c) limited ability to attract highly academic faculty, d) more modest laboratory and research facilities and e) smaller physical space. Many other differences may be present; we have presented some of the obvious dissimilarities. However, our school was able to offset some of our road-blocks by using our funds judiciously and sell the advantages of a smaller school located in a salubrious environment. Understandably, we could not get around many of the difficulties universal to new schools. In general, the small faculty is more collegial, optimistic and of the view that not all the Goliaths win the battle.

In the personal category, there were a few areas of dissatisfaction. Overall, as expected, the faculty were happy with their rank and salaries, professional growth opportunities and health benefits. There was some dissatisfaction with retirement benefits, which may not be universally valid especially since retirement benefits had not yet been put into place. An absent tenure-track system was a source of dissatisfaction which again, was intuitively anticipated. The 2015 COACHE Faculty Satisfaction Survey showed comparable results [17].

In the Support category, there was satisfaction with the leadership and the style of management. However, this could vary at different institutions. One source of pride and satisfaction for the faculty was opportunities to take on new administrative leadership roles (e.g. assistant dean of student affairs/curriculum development, etc.) which was unlikely to be available in established medical schools. Also, there was better support from colleagues. Since everyone was new, there were negligible social clustering or grouping which is often present in more established schools. One recurring problem area was mentorship. Over half the faculty (58%) seemed unsatisfied by the mentoring opportunities. Many faculty felt that there was a lack of mentoring for their guidance and advancement. However, this was counter balanced by unique opportunities to mentor medical and graduate students. The faculty being few, more students were available per faculty; hence, academic advising of medical students became an attractive proposition in the new medical school. In addition, ample opportunities to participate in the governance of the new school were highly valued by the founding faculty. A source for concern in this category was poor system in place for conflict resolution, a problem that should be resolved in the near future.

A new medical school like CUSM - SOM is attractive to its faculty for many reasons. Their professional growth is better, since it allows more autonomy with less bureaucracy and is easier to implement one’s ideas. Furthermore, to attract faculty from well-established medical institutions, a new school may provide better salaries and offer higher academic ranks and administrative positions. However, in new schools there are unanticipated new challenges which may impact long term retention. Awareness of these issues will help the new institutions as well as the newly recruited faculty to find lasting solutions.

There were many avenues of satisfaction and pride working for a new medical school. The interaction between colleagues was excellent; this was anticipated as the new faculty were forging new relationships and seeking to build bridges with their colleagues. This was also because few members of the faculty had to work closely towards developing the curriculum, which delivers basic science concepts linked to 90 important clinical presentations integrated across disciplines (e.g. Biochemistry, Anatomy, Microbiology, etc.) and across years (e.g. Medicine, Surgery, Psychiatry, etc.). Hence, termed horizontal and vertical integration respectively. The curriculum is aptly named “Clinical Presentation Driven and Active Learning”.

The technical support was perceived to be outstanding as were the working hours. There was some ambivalence about gender diversity since there were few female faculty. This was not by design but circumstantial. The number of female applicants was significantly lower than male applicants, with a F:M ratio of applications received being 1: 3. One area of dissatisfaction was opportunities for promotion and another area being support for scholarly activities. These were teething problems to be expected in any new medical school. As indicated in 2015 COACHE Faculty Satisfaction Survey [17], a key factor for high rating of the review and promotion process was the clarity of the process itself. As an medical school awaiting medical school, such processes are not well developed and need further improvement. The current promotion process evaluates faculty performance in 3 areas: scholarly activities (e.g. research productivity, grant proposals, patents, etc.), teaching skills and service efficiency; two of three categories have to be outstanding for promotion. Even a faculty applying for promotion based on teaching and service efficiency is required to maintain baseline scholarly functions with a minimum of 5 peer-reviewed research publications over 5 to 6 years in their respective rank.

The faculty members also felt that administration was not taking enough measures to retain valued faculty. This concern of the faculty is consistent with another study performed at University of Colorado Medical School, where clinical faculty turnover was 34% within the first 3 years of hiring [10]. In this study, numerous factors were identified for early attrition of the faculty: not rewarding excellence, non-fostering of teaching, research and creativity. These findings are inconsistent with a similar study on early attrition, where the indicators were early faculty dissatisfaction and misaligned expectations rather than academic success or promotion [10]. Our results are also consistent with the 2012 study performed by the Pololi et al. [11], where 21% of the faculty wanted to leave academic medicine because of dissatisfaction and low intuitional support. Yet another study showed that among 80% of the faculty, lack of satisfaction of their work being appreciated, seemed a major factor in intention to leave the institution [7].

The small number of faculty participating in the survey of our new medical school is a limitation of this study. Another limitation was that there were few junior faculty in the survey. However, with the evolution of the school, more junior faculty have joined since this survey. Follow-up studies will address the needs for junior faculty for retaining and promoting them [18].

However, the aims of this study were modest and in no way all-encompassing. Also, many questions were general and could not be categorized into a specific theme. For example, poor opportunities for promotion could be institutional as well as personal.

## Conclusions

This study underscores the unique issues facing the faculty in a new medical school. Based on this study, to retain the founding faculty a) institutional and individual faculty development programs were developed, b) a “learning community” to foster collegiality (both internally and externally) was instituted, and c) the leadership was cautioned to be more attuned to the needs of the founding faculty.

In conclusion, a new medical school offers unique opportunities to its founding faculty. It fosters new leadership opportunities, helps to forge new relationships and offers a fertile ground for faculty development. If the few roadblocks are carefully resolved, new medical schools can attract outstanding faculty from well-established schools worldwide.
